# Abnormal upregulation of NUBP2 contributes to cancer progression in colorectal cancer

**DOI:** 10.1007/s11010-024-04956-8

**Published:** 2024-03-16

**Authors:** Danfeng Lan, Junyu Wang, Guishun Sun, Lixia Jiang, Qiyun Chen, Sha Li, Haiyan Qu, Yibo Wang, Bian Wu

**Affiliations:** 1 Department of Gastroenterology, The First People’s Hospital of Yunnan Province, The Affiliated Hospital of Kunming University of Science and Technology, Kunming, 650032 Yunnan China; 2 Department of General Surgery II, The First People’s Hospital of Yunnan Province, The Affiliated Hospital of Kunming University of Science and Technology, No. 157, Jingbi Road, Kunming, 650032 Yunnan China

**Keywords:** Colorectal cancer, NUBP2, GSK3β, Malignant phenotype, Tumor growth

## Abstract

Colorectal cancer (CRC), a digestive tract malignancy with high mortality and morbidity, lacks effective biomarkers for clinical prognosis due to its complex molecular pathogenesis. Nucleotide binding protein 2 (NUBP2) plays a vital role in the assembly of cytosolic Fe/S protein and has been implicated in cancer progression. In this study, we found that NUBP2 was highly expressed in CRC by TCGA database analysis. Subsequently, we verified the expression of NUBP2 in CRC tumor tissues and para-carcinoma tissues using IHC staining, and further investigated its association with clinicopathological parameters. In vitro cell experiments were conducted to assess the role of NUBP2 in CRC by evaluating cell proliferation, migration, and apoptosis upon NUBP2 dysregulation. Furthermore, we established a subcutaneous CRC model to evaluate the impact of NUBP2 on tumor growth in vivo. Additionally, we performed mechanistic exploration using a Human Phospho-Kinase Array-Membrane. Our results showed higher expression of NUBP2 in CRC tissues, which positively correlated with the pathological stage, indicating its involvement in tumor malignancy. Functional studies demonstrated that NUBP2 knockdown reduced cell proliferation, increased apoptosis, and impaired migration ability. Moreover, NUBP2 knockdown inhibited tumor growth in mice. We also observed significant changes in the phosphorylation level of GSK3β upon NUBP2 knockdown or overexpression. Additionally, treatment with CHIR-99021 HCl, an inhibitor of GSK3β, reversed the malignant phenotype induced by NUBP2 overexpression. Overall, this study elucidated the functional role of NUBP2 in CRC progression both in vitro and in vivo, providing insights into the molecular mechanisms underlying CRC and potential implications for targeted therapeutic strategies.

**Supplementary Information:**

The online version contains supplementary material available at 10.1007/s11010-024-04956-8.

## Introduction

Colorectal cancer (CRC) is a common digestive tract malignancy contains colon and rectal cancer [1]. According to statistics, in 2020, there are approximately 1.93 million new cases, 916 000 deaths from CRC in the world annually (https://www.who.int/news-room/fact-sheets/detail/cancer), imposing a huge economic burden on the whole society.

Surgery is the standard treatment for early stage CRC [2]. Whereas most patients are diagnosed with CRC at an advanced stage, by which metastasis has occurred and eventually succumb to the disease [3]. As therapeutic technology, including surgery combined with chemotherapy or radiotherapy has progressed, many breakthroughs have been made in the treatment of CRC [4]. Regrettably, tumor recurrence, distant metastasis, drug resistance and toxic effects are still a worldwide difficult for the clinician. Over the past dozen years, many molecules associated with cancer have been identified, and targeting these biomarkers are essential for the development of targeted therapy in metastatic CRC [5–7]. However, the clinical outcomes were not approving enough, and the effective biomarkers for the clinical prognosis of CRC remain to be widely investigated due to its heterogeneous and intricate molecular pathogenesis.

Nucleotide binding protein 2 (NUBP2), located in the t-complex region of mouse Chromosome 17, is a conserved and vital protein in Eukaryotes [8]. NUBP1 and NUBP2 play a crucial role in the cell cycle as integral components of centrioles and are involved in controlling centrosome duplication in mammalian cells [8, 9]. Moreover, NUBP2 is a key component of the cytosolic iron–sulfur (Fe/S) protein assembly, which is essential for the fundamental metabolism of all organisms [10]. A previous study demonstrated that NUBP2 is necessary for embryogenesis [11]. In Barrett’s esophagus with high-grade dysplasia, suppressing NUBP2 significantly impacts cell morphology [12]. Furthermore, RNA-sequencing analysis identified it as a potential biomarker for diagnosing sarcopenia [13]. Aberrant NUBP2 expression has also been observed in several cancers, including gastric cancer [14] and hepatocellular carcinoma [15]. However, the functional role of NUBP2 in CRC remains unclear.

In the current study, the expression patterns of NUBP2 in the CRC tissues and cell lines were firstly compared with the normal tissues and cells. Subsequently, in vitro functional assays were performed to verify the biological role of NUBP2 in the regulation of CRC cell malignant behaviors through NUBP2 knockdown. Additionally, the effects of NUBP2 knockdown on the tumor growth in vivo were investigated in a CRC xenograft model. These findings provided evidence supporting the potential of NUBP2 as a target for the development of novel therapeutic interventions against CRC.

## Materials and methods

### Cell lines, tissue microarray, and animals

Human CRC cell lines RKO, HT29 and HCT 116, as well as human normal colonic epithelial cells FHC, were obtained from ATCC and cultured in 1640 medium supplemented with 10% fetal bovine serum (FBS). All cell lines were kept in a 37 °C incubator with 5% CO_2_.

A tissue microarray generated from 114 CRC tissues and 94 normal para-carcinoma tissues was provided by Shanghai Yibeirui Biomedical Science and Technology Co., Ltd. (Shanghai, China). The patients’ clinical baseline data were collected with their written informed consent.

Four-week-old female BALB/c nude mice were obtained from Gempharmatech Co., Ltd. in Jiangsu, China. The mice were housed in groups of five per cage under controlled conditions, including a temperature of 22–25 °C, humidity of 50–60%, and a 12-h light/dark cycle. Ample food and water were provided to the mice.

### Immunohistochemistry (IHC) staining

Section samples were dewaxed in xylene and then rehydrated through graded ethanol (100%, 95%, 85% and 75%). The sections were washed in flowing water and subjected to antigen retrieval with using citrate buffer (pH 6.0) before blocking endogenous peroxidase activity with 3% H_2_O_2_ for 5 min. Subsequently, the sections were blocked with 5% normal serum and incubated with primary antibodies (NUBP2, 1:100, Sanying, #15409-1-AP; Ki-67, 1:100, Abcam, #ab16667) overnight at 4 °C. After washing, secondary antibody (goat anti-rabbit IgG H& L (HRP): 1:400, Abcam, #ab97080) was added for incubation of sections. Afterwards, DAB coloration and hematoxylin counterstaining were performed (Baso Diagnostics Inc., Zhuhai, China). The slides were sealed with neutral resin, and images were captured under microscope. The scoring system takes into account both the level and the amount of staining. The IHC scoring method was used to evaluate the images, using a scale that ranges from negative (0), positive (1–4), ++ positive (5–8), to +++ positive (9–12), as has been previously described [16].

### Establishment of stably infected cells

Three different short hairpin (shNUBP2-1: 5′-GATGGGAATCGTGGAGAATAT-3′, shNUBP2-2: 5′-GCGAGCTGACCTTCTGTAGGA-3′, and shNUBP2-3: 5′-GCCACCATAGAAGCCCTGCGT-3′) were used to specifically targeting NUBP2 gene. A randomly rearranged sequence, 5′-TTCTCCGAACGTGTCACGT-3′, was utilized as a negative control for shRNA (shCtrl). After annealing, the double-stranded DNA oligos were joined with a linearized vector through T4 DNA ligase. The resulting recombinant vector was then transformed into competent Escherichia coli cells and cultured in LB medium supplemented with ampicillin at 37 °C. The plasmid was extracted using the EndoFree Maxi Plasmid Kit (TIANGEN, China) following the manufacturer’s instructions. For lentivirus production, plasmids, pMD2.G (Qiagen, China), and psPAX2 (Qiagen, China) were transfected into 293T cells using Lipofectamine^®^ 2000 (Thermo Fisher, USA).

Seeding cells in 6-well plates at a density of 5 × 10^4^ cells per well and incubating them at 37 °C with 5% CO_2_. The lentivirus vector or control virus was added to the culture when the cells reached 30–50% confluency and incubated for 24 h. Subsequently, puromycin (2 μg/mL) was introduced to the medium to select and maintain the infected cells for further experiments.

### Quantitative real-time PCR (qRT-PCR)

The cells were used to isolate Total RNA, by following the manufacturer’s protocol, with the TRIzol^®^ reagent (Sigma, USA). The purity and concentration of the RNA extracted were evaluated by Nanodrop 2000/2000C spectrophotometry (Thermo, USA). Next, the cDNA was synthesized using the Hiscript QRT supermix according to the manufacturer’s instructions (Vazyme, China). qRT-PCR performed using SYBR Green mastermix (Vazyme, China). For normalization purposes, GAPDH was utilized as an internal control. The 2^−ΔΔCt^ method was employed to determine the relative expression levels. The primers sequences (5′–3′) were listed as follows: the forward primer of NUBP2 is GTGGAGAGGCCCCAAGAAAA, the reverse primer is TAGGGACGCAGGGCTTCTAT; the forward primer of GAPDH is TGACTTCAACAGCGACACCCA, the reverse primer is CACCCTGTTGCTGTAGCCAAA.

### Western blot analysis

The cells were lysed with lysis buffer on ice for a period of 30 min. The concentration of proteins was determined using the BCA protein assay kit (HyClone-Pierce, USA). Later, the proteins were separated using 10% SDS-PAGE and transferred to PVDF membranes. Subsequently, the membranes were blocked with fat-free milk (5%) at room temperature for 1 h, and then exposed to primary antibodies overnight at 4 °C. The next day, the PVDF membranes were treated with secondary antibodies at room temperature for 1 h. After being washed with PBS three times, the protein bands were detected using the immobilon Western Chemiluminescent HRP Substrate kit (Millipore, USA). The antibodies used in Western blot analysis are shown in Table S1.

### Celigo cell counting assay

For the Celigo cell counting assay, HCT 116 and RKO cells infected with shCtrl and shNUBP2 were seeded in 96-well plates with a density of 2 × 10^3^ cells per well and incubated at 37 °C with 5% CO_2_. The Celigo system (Nexcelom) was employed to determine the cell number for 5 consecutive days. The data obtained was subjected to statistical analysis to generate a 5-day cell growth curve.

### Colony formation assay

In the colony formation assay, 2000 cells/well were seeded in a 6‐well dish after being transfected for 48 h, and then cultured for 8 days until visible colonies formed. Afterwards, the cells were fixed in 4% paraformaldehyde, stained with crystal violet at room temperature for 30 min, and then the cell colonies were counted microscopically. Any shaking or moving with plates was avoided to obtain clear colonies. All experiments were performed in triplicate and repeated three times.

### Flow cytometry

HCT 116 and RKO cells infected with lentivirus were cultured in 6-well plates at 37 °C. When the cells reached 85% confluence, the cells were harvested and washed with 4 °C D-Hanks solution (pH 7.2–7.4) after centrifugation at 1300 rpm. Subsequently, the cells were suspended in 200 μL of 1× binding buffer and stained with 10 μL of Annexin V-APC (#88-8007, eBioscience, China) in the absence of light for 15 min, followed by the addition of 5 μL propidium iodide (PI) solution. Cell apoptosis was assessed using the Guava easyCyte HT flow cytometer (Millipore, USA).

### CCK8 assay

HCT 116 and RKO cell lines were digested, resuspended, and counted in the absence or presence of CHIR-99021 HCl (CHIR, 10 μM, SF2703-25 mg), a GSK3 inhibitor. 100 μL cell suspension was added to each well of a 96-well plate at a density of 3000 cells per well, followed by incubation for 24 h. Starting from the second day, CCK-8 reagent (10 μL) was added to each well 4 h before the end of the incubation period. After that, the 96-well plate was shaken for 2–5 min on a shaker, and the optical density (OD) was measured using a microplate reader at 450 nm for 5 consecutive days. The experiment was repeated three times.

### Transwell assay

Transwell migration assays were performed to assess migration and invasion of HCT 116 and RKO cells using Transwell plates according to the the manufacturers instructions (Corning). For the cell invasion assay, the upper chamber was coated with Matrigel, whereas non-coated chamber was used for the cell migration assay. The cells in logarithmic growth period were cultured under starvation condition for 12 h and then digested by trypsin, washed by PBS for 1–2 times, resuspended in serum-free medium. Cells suspended in serum-free medium (1 × 10^5^ cells) were seeded to the top chamber after 600 μL RPMI-1640 medium with 10% FBS was added to bottom chamber. After 12 h, non-migrated cells were removed using a cotton swab, while the migrated cells adhering to the lower surface of the membrane were fixed and stained with crystal violet at 25 °C for 3 min. Following washing with PBS, cells were imaged using a 400× microscope in three random fields and quantified using ImageJ software (National Institutes of Health, version 1.8.0).

### Mouse xenograft tumor assay

In order to establish xenograft models, BALB/c nude mice were subcutaneously injected in the right flank with RKO cells infected with shNUBP2 or shCtrl lentivirus (at a density of 1 × 10^7^ cells/mice). The mice were then divided into shNUBP2 and shCtrl groups, respectively (*n* = 6). The size of the tumors was measured to calculate the tumor volume using the formula (tumor volume = *π*/6 × *L* × *W* × *W*). After 22 days of injection, the mice were sacrificed, and the tumors were excised, weighed, and analyzed for further analysis.

### Statistical analysis

Data analysis was performed using GraphPad Prism 7. The data was presented as mean ± standard deviation (SD). Analysis of variance (ANOVA) or the unpaired *t* test for two-group comparisons was used for statistical analysis. The experiments were conducted in triplicate and repeated at least three times. The Mann Whitney *U* test and Spearman’s rank correlation analysis were applied to investigate the correlation between the expression of NUBP2 and tumor characteristics in patients with CRC. A *P* value lower than 0.05 was deemed statistically significant.

## Results

### Upregulation of NUBP2 in CRC tissues and cells

**Fig. 1:**
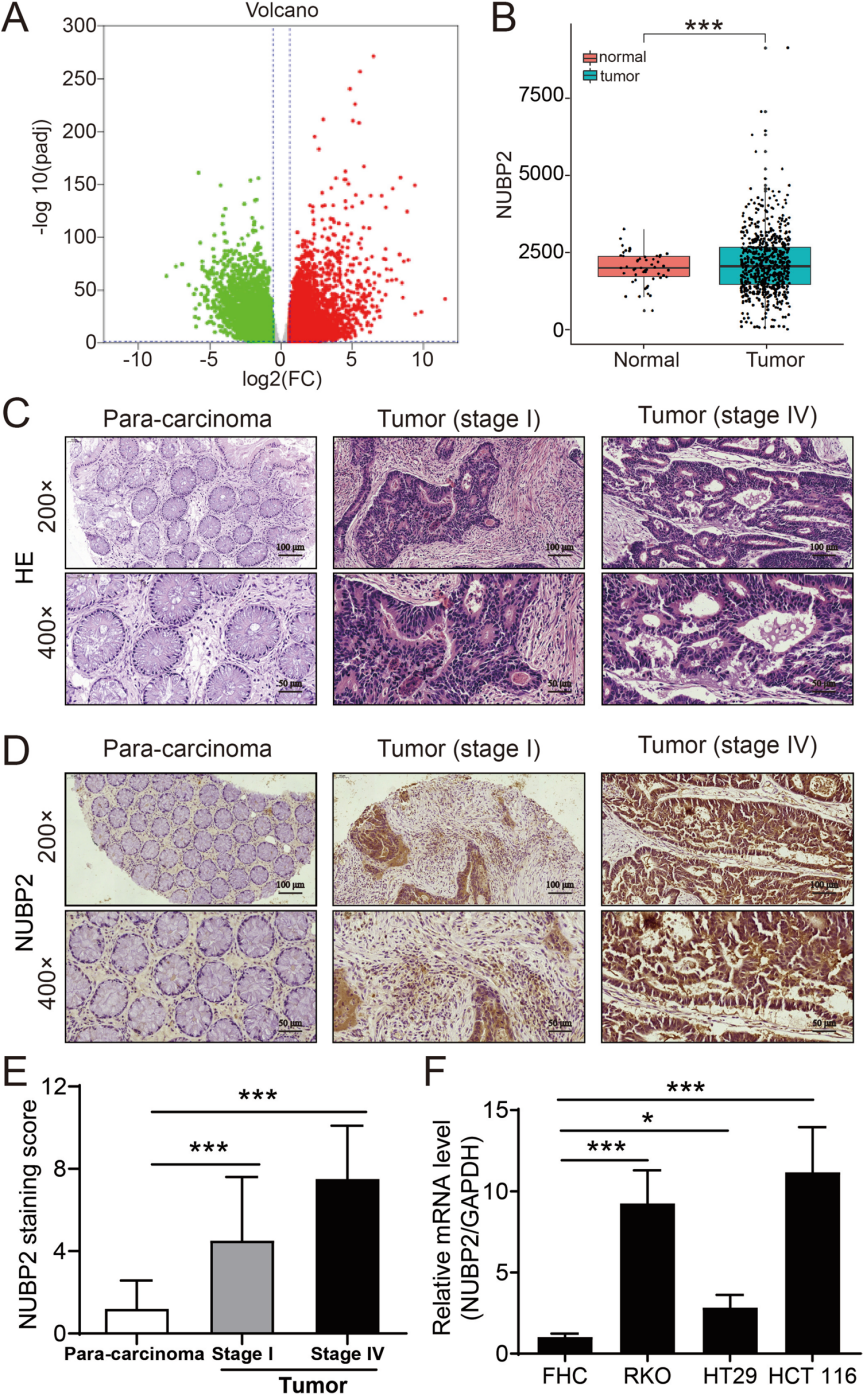
NUBP2 was highly expressed in CRC tissues and cells. **A** Volcano plot for differential gene expression. **B** Comparison of NUBP2 expression levels in CRC tissues and normal tissues using TCGA RNA-seq data. **C** HE stain performed on the tissue microarray. **D** IHC staining results for the positivity of NUBP2. **E** Quantification of IHC staining results. **F** Assessment of NUBP2 mRNA expression in CRC cell lines (RKO, HT29 and HCT116) compared to the normal colon epithelial cells FHC. Results are presented as means ± SD. *P < 0.05 and ***P < 0.001

To characterize the expression of NUBP2 in CRC, we firstly analyzed the RNA sequencing (RNA-seq) data of CRC tissues from TCGA. It was found that NUBP2 expression was elevated in CRC tissues compared to normal tissues (Fig. 1A, B). Subsequently, we performed histopathological examinations using hematoxylin and eosin (HE) and IHC staining on a tissue microarray containing CRC tumor tissues and para-carcinoma tissues. HE staining was employed to assess the pathological features of CRC tissues, revealing irregular nuclear morphology, intense chromatin staining, and heterogeneous cytoplasm, whereas para-carcinoma tissues exhibited normal nuclear morphology, uniform staining (Fig. 1C). Consistently, IHC staining of NUBP2 validated the strong positive expression of NUBP2 was in CRC tissues, with particularly enhanced expression observed in tissues from stage 4 (Fig. 1D, E). Statistical analysis demonstrated that 55/114 (48.2%) of CRC tissues exhibited high NUBP2 expression, while only 1/94 (1.1%) in para-cancerous tissues (*P* < 0.001) (Table 1). By analyzing the clinicopathological data, it was revealed that the expression of NUBP2 was related to metastasis and tumor stage (*P* < 0.05 for both) (Tables 2, 3). Additionally, we further verified the expression level of NUBP2 at the cellular level by RT-qPCR. As shown in Fig. 1F, the expression levels of NUBP2 in human CRC cell lines RKO, HT29 and HCT 116 were higher than the human normal colonic epithelial cells FHC. Collectively, these results implied that NUBP2 has an important role in CRC progression.

**Table 1:** Expression patterns in colorectal cancer tissues and para-carcinoma tissues revealed in immunohistochemistry analysis

NUBP2 expression	Tumor tissue		Para-carcinoma tissue		*P* value
	Cases	%	Cases	%	
Low	59	51.8	93	98.9	< 0.001
High	55	48.2	1	1.1

**Table 2:** Relationship between NUBP2 expression and tumor characteristics in patients with colorectal cancer

Features	No. of patients	NUBP2 expression		*P* value
		Low	High	
All patients	114	59	55	
Age (years)				0.995
< 58	56	29	27	
≥ 58	58	30	28	
Gender				0.157
Male	69	32	37	
Female	45	27	18	
Tumor size				0.714
≤ 4.7 cm	58	31	27	
> 4.7 cm	56	28	28	
Stage				0.027
0	2	2	0	
I	4	3	1	
II	63	35	28	
III	40	19	21	
IV	5	0	5	
Lymphatic metastasis (N)				0.350
N0	73	40	33	
N1	31	15	16	
N2	10	4	6	
Lymph node positive (individual)				0.816
= 0	82	43	39	
> 0	32	16	16	
Metastasis				0.018
M0	109	59	50	
M1	5	0	5

**Table 3:** Relationship between NUBP2 expression and tumor characteristics in patients with colorectal cancer

		NUBP2
Metastasis	Spearman correlation	0.222
	Significance (two-tailed)	0.018
	*N*	114
Stage	Spearman correlation	0.208
	Significance (two-tailed)	0.027
	*N*	114

### NUBP2 was successfully knocked down in CRC cells

**Fig. 2:**
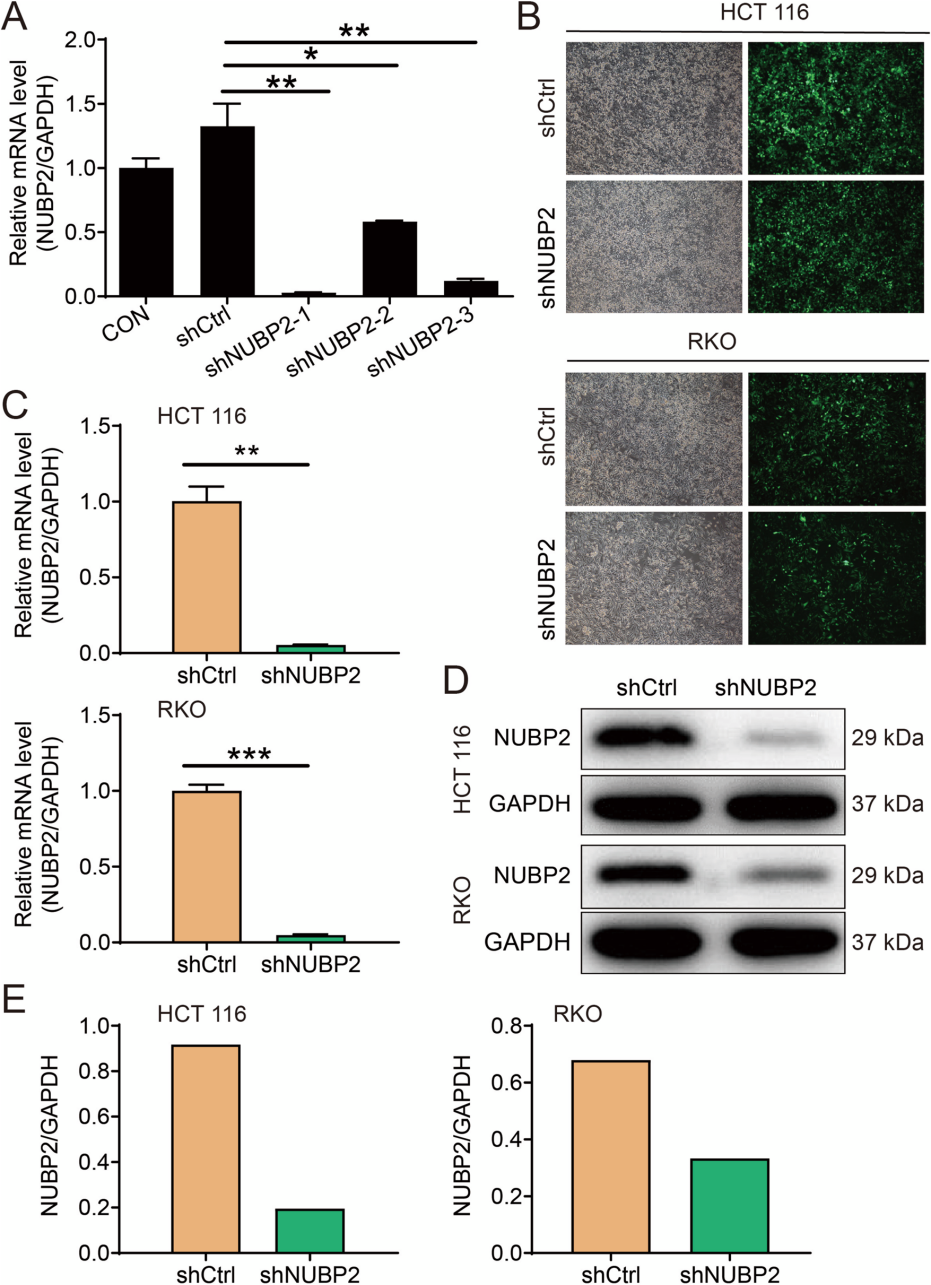
Construction of NUBP2 knockdown cell models in CRC cells. **A** Three short hairpin RNA (shRNA) sequences were screened by RT-PCR. **B** Successful infection of cells was confirmed by the presence of green fluorescent protein (GFP) fluorescence. **C, D** Knockdown efficiency of NUBP2 in HCT 116 and RKO cells was assessed using RT-qPCR (**C**) and western blot (**D**) assays. **E** Quantification data of protein bands. Results are presented as means ± SD. *P < 0.05; **P < 0.01 and ***P < 0.001

To thoroughly survey the roles of NUBP2 in CRC, NUBP2 knockdown cell models were constructed in CRC cells by transfecting shNUBP2 or shCtrl lentiviral vector. It was found that the mRNA level of NUBP2 expression in HCT 116 cells was dramatically reduced in shNUBP2 groups compared to the shCtrl group, and shNUBP2-1 with higher knockdown efficiency (97.8%) was selected for use in the subsequent experiments (Fig. 2A). The fluorescence microscopic results indicated that HCT 116 and RKO cells were successfully infected by the lentivirus (Fig. 2B). Subsequently, we detected the knockdown efficiency of NUBP2 in HCT 116 and RKO cells by RT-qPCR and western blot assays. When compared with the shCtrl group, the expression of NUBP2 was decreased in shNUBP2 group at both mRNA and protein levels (Fig. 2C–E). Overall, these results indicated that NUBP2 was successfully knocked down in two shNUBP2 transfected CRC cell lines.

### Loss of NUBP2 expression suppressed the malignant behaviors of CRC cells

**Fig. 3:**
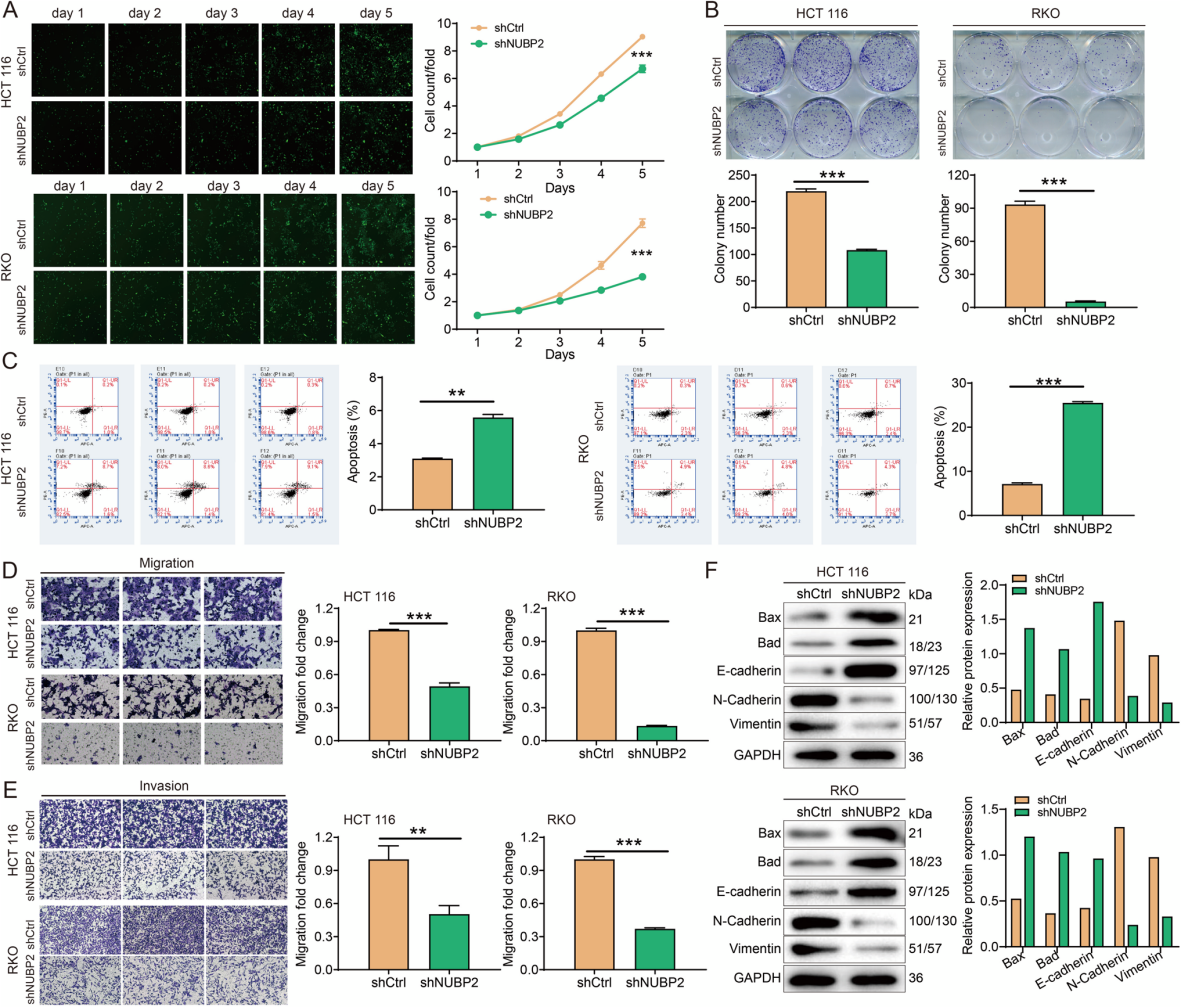
NUBP2 regulated the malignant behavior of CRC cells. **A** Cell proliferation in HCT116 and RKO cell lines was evaluated using Celigo cell counting assay upon NUBP2 knockdown, and photomicrographs were captured at a ×100 magnification. **B** The cloning abilities of HCT116 and RKO cells with or without NUBP2 deletion were detected using clone formation assay. **C** Apoptosis rates of HCT116 and RKO cells were examined by flow cytometry following NUBP2 knockdown. D-E, Transwell assays were employed to measure the effect of NUBP2 knockdown on cell migration ability (**D**) and invasion ability (**E**) of both CRC cell lines. Representative images were captured at magnifications of ×200 for migration and ×100 for invasion, respectively. F, Western blot analysis was performed to assess the apoptotic markers and EMT markers after NUBP2 knockdown. Results are presented as means ± SD. **P < 0.01 and ***P < 0.001

Next, the effects of NUBP2 on proliferation, clone formation, apoptosis, and migration were evaluated in CRC cells upon NUBP2 silencing. Celigo cell counting assay indicated that the proliferation rates of HCT 116 and RKO cell lines were slower in shNUBP2 group than the shCtrl group (*P* < 0.001 for both) (Fig. 3A). Similarly, the colony formation efficiency was apparently weakened in HCT 116 and RKO cell lines upon NUBP2 depletion (*P* < 0.01, *P* < 0.001) (Fig. 3B). The results of flow cytometry revealed that NUBP2 knockdown significantly accelerated the apoptosis rates of HCT 116 and RKO cells compared to the shCtrl group (*P* < 0.001 for both) (Fig. 3C). Furthermore, transwell assay showed that the migration rates of HCT 116 and RKO cells in shNUBP2 group were decreased by 50% and 87%, respectively (*P* < 0.001 for both) (Fig. 3D), suggesting that NUBP2 deletion potently impaired the migratory capacity of CRC cells. Consistently, invasion of both CRC cell lines were also decreased under NUBP2 knockdown by the Transwell assay (Fig. 3E). Altogether, these results provided evidence for the role of NUBP2 in regulating the proliferation, apoptosis, migration and invasion of CRC cells.

Moreover, the expression levels of pro-apoptotic proteins Bax and Bad were increased in shNUBP2 cells compared to the shCtrl cells (Fig. 3F). Additionally, Western blot analysis demonstrated downregulation of epithelial-mesenchymal transition (EMT) biomarkers N-Cadherin and Vimentin, while upregulation of E-cadherin upon NUBP2 knockdown (Fig. 3F). These results further validated that NUBP2 silencing suppressed the malignant behaviors of CRC cells in vitro.

### Downregulation of NUBP2 suppressed tumor growth in CRC mice

**Fig. 4:**
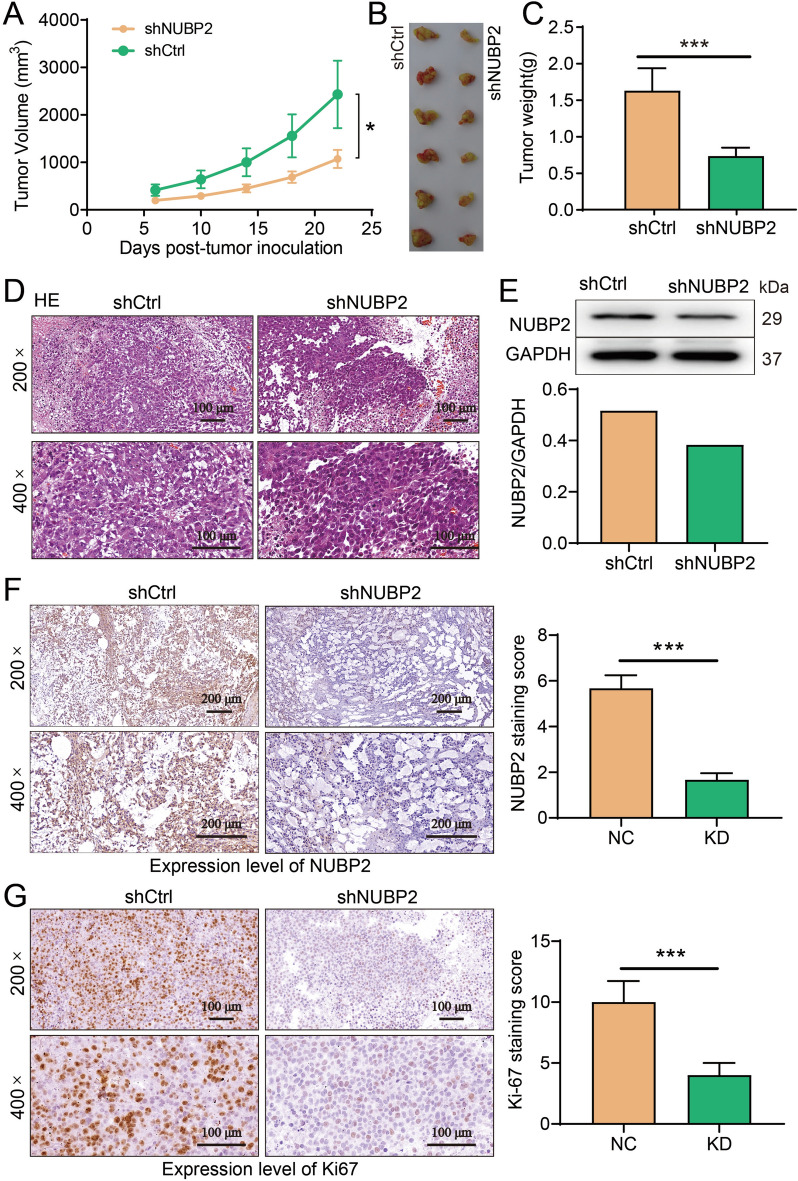
Silencing of NUBP2 suppressed CRC xenograft tumor growth in mice. **A** The tumor volume was calculated to determine the tumor growth rates. **B** Tumor size was monitored with calipers. **C** Tumor weight of mice in the shCtrl group and shNUBP2 group was measured. **D** The tumor tissues from nude mice were subjected to HE staining. **E** NUBP2 protein levels in tumor tissues of mice between the two groups were measured by western blot analysis. **F** IHC staining of NUBP2 in mice tumor tissue. **G** Ki67 staining of tumor tissue were performed to assess cell proliferation in vivo. Results are presented as means ± SD. *P < 0.05 and ***P < 0.001

To validate the suppressive effect of NUBP2 knockdown on CRC tumor growth in vivo, a mouse xenograft model was established using RKO cells with or without NUBP2 knockdown. The tumor growth curves revealed that NUBP2 knockdown significantly retarded the growth of CRC tumors in mice compared to the mice of shCtrl group (Fig. 4A). After 30 days, all mice were sacrificed, and tumors were excised. It was found that the tumors formed in the shNUBP2 group of mice were smaller than those in the shCtrl group (Fig. 4B). Consistently, the tumor weight of the CRC mice in shNUBP2 group was significantly lower than that of the shCtrl group (Fig. 4C). HE staining showed a higher incidence of necrotic areas with cell body breakdown in the shCtrl group, while the shNUBP2 group exhibited organized cells and typical cellular structure (Fig. 4D). Besides, the expression of NUBP2 protein in the tissues from shNUBP2 mice was substantially downregulated compared to those in the tissues of shCtrl mice (Fig. 4E, F). These results indicated that effectively downregulating NUBP2 resulted in the suppression of tumor growth in CRC mice. In order to assess the biological significance of NUBP2 on tumor cell proliferation, IHC staining for Ki-67 (an indicator of proliferation) were performed in tumor tissues of each group. It could observe that positive expression of Ki-67 in the tissues from shNUBP2 mice was significantly weaker than the shCtrl mice tumors (Fig. 4G). Collectively, these data strongly demonstrated that downregulation of NUBP2 repressed CRC tumor progress in vivo.

### NUBP2 exert critical roles in CRC progression via regulation of GSK-3β

**Fig. 5:**
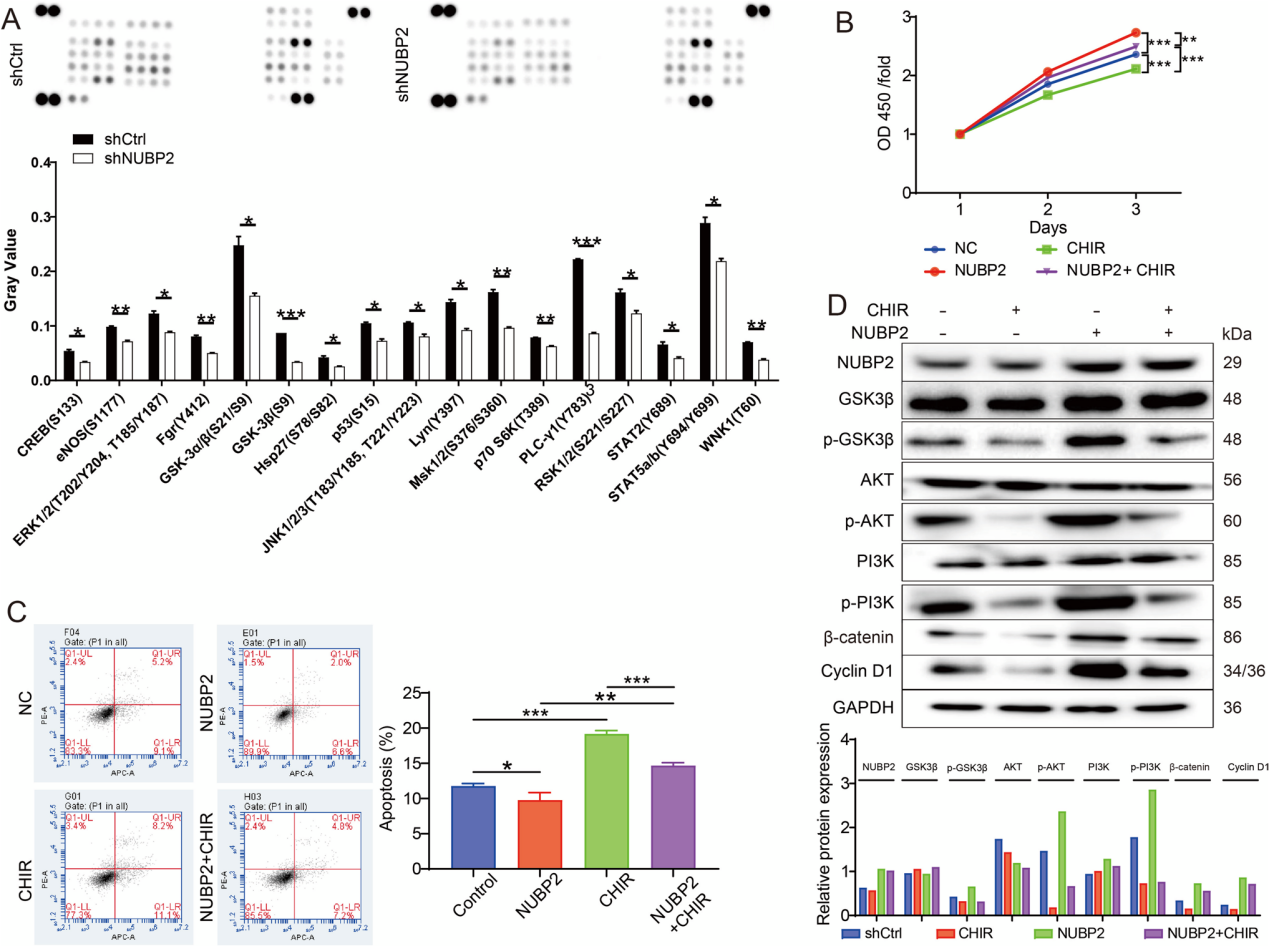
NUBP2 exert critical roles in CRC progression via regulation of GSK-3β. **A** A human phospho-kinase array was performed in RKO cells upon NUBP2 knockdown. **B** CCK8 assay was performed to assess the proliferation of RKO cells with NUBP2 overexpression or CHIR-99021HCl (a GSK3 inhibitor) treatment. **C** The effect of NUBP2 overexpression on RKO cells apoptosis was assessed by flow cytometry in the presence or absence of CHIR-99021HCl. **D** The expression of NUBP2, GSK-3β, AKT, PI3K, β-catenin and Cyclin D1 was analyzed by western blot in RKO cells with NUBP2 overexpression and CHIR-99021HCl treatment. Results are presented as means ± SD. *P < 0.05, **P < 0.01 and ***P < 0.001

In order to elucidate the underlying molecular mechanisms of NUBP2 in CRC progression, a human phospho-kinase array was performed in RKO cells upon NUBP2 knockdown, which could identify the essential pathway kinases that were influenced. It was observed that NUBP2 knockdown reduced the phosphorylation levels of CREB (S133), eNOS (S1177), ERK1/2 (T202/Y204, T185/Y187), Fgr (Y412), GSK-3α/β (S21/S9), GSK-3β (S9), Hsp27 (S78/S82), p53 (S15), JNK1/2/3 (T183/Y185, T221/Y223), Lyn (Y397), Msk1/2 (S376/S360), p70 S6K (T389), PLC-γ1 (Y783), RSK1/2 (S221/S227), STAT2 (Y689), STAT5a/b (Y694/Y699) and WNK1 (T60) compared to the shCtrl group (Fig. 5A). Next, we focused on GSK-3 in the following study, as it underwent the most significant down-regulation. The GSK3 inhibitor CHIR-99021 HCl was then used to treat CRC cells and assessed its effects on cell phenotypes in CRC cells with NUBP2 overexpression. Similar to NUBP2 deletion, GSK3 inhibitor CHIR-99021 HCl (10uM) inhibited proliferation and promoted apoptosis (Fig. 5B, C). On contrary, overexpression of NUBP2 had the opposite effects (Fig. 5B, C). Surprisingly, the pro‐oncogenic effects of NUBP2 were significantly weakened by CHIR-99021 HCl. Collectively, these findings underscored the pivotal role of NUBP2 in regulating GSK-3, thereby influencing the phenotype of CRC cells.

Previous studies have demonstrated that the PI3K/AKT/GSK3β signaling pathway is implicated in the EMT and progression of CRC [17, 18]. Our investigation also demonstrated that knocking down NUBP2 effectively inhibited EMT in CRC (Fig. 3F). Furthermore, western blot analysis revealed an increase in the levels of phosphorylated GSK-3β, AKT and PI3K, as well as the protein levels of β-catenin, Cyclin D1 in RKO cells overexpressing NUBP2, which was partially reversed upon treatment with GSK3 inhibitor CHIR-99021 HCl (Figs. 5D and S1), implying that NUBP2 might regulate CRC EMT through PI3K/AKT/GSK3β signaling.

## Discussion

CRC is a common malignant tumor, characterized by high incidence rate, metastasis rates and mortality, as well as poor prognosis [19]. Recently, a considerable body of evidence has implicated that identification of key molecular biomarker could improve molecular diagnosis and treatment of patients with CRC patients. Morever, NUBP2 has been found to disturb Fe–S cluster biogenesis and is responsible for regulating centrosome duplication and ribosome biogenesis in yeast [20]. Here, higher expression of NUBP2 was observed in CRC tissues and cell lines compared to para-carcinoma tissues and normal cells, and this elevated expression was associated with increased tumor malignancy. Therefore, this work focused on exploring the role NUBP2 plays in CRC.

NUBP2, a member of nucleotide-binding proteins (NUBP) [21], is involved in various physiological functions. Together with NUBP1, NUBP2 plays a critical role in the maturation of cytosolic and nuclear Fe–S proteins [22]. It has been discovered that the production of long splicing isoform of NUBP2 suppressed cancer cell proliferation in oral squamous cell carcinoma [23]. On the other hand, NUBP2 is required for cytosolic Fe/S protein assembly and cellular iron metabolism, and its knockdown leads to reduced cell viability [24]. Consistently, our results demonstrated that NUBP2 knockdown resulted in suppressed cell proliferation and enhanced cell apoptosis in CRC cells. Whereas overexpressing NUBP2 promoted cell proliferation and suppressed apoptosis. Correspondingly, animal xenografts demonstrated that loss of NUBP2 resulted in a decreased tumor growth in vivo. The EMT is a biological process that contributes to the acquisition of mesenchymal cell features, and it has been demonstrated to increase invasiveness and migratory capabilities of CRC cells [25, 26]. Notably, the current study revealed that depleting NUBP2 effectively inhibited cell migration and invasion. This was confirmed by suppressing EMT through reducing N-cadherin and Vimentin expression, and increasing E-cadherin expression. These findings collectively indicated that NUBP2 plays a crucial role in promoting cancer development in CRC. Next, we attempted to preliminarily unveil the potential molecular mechanism by which NUBP2 regulated CRC progression.

Glycogen synthase kinase 3 beta (GSK3β) plays a crucial role in various biological processes such as cellular metabolism, cytoskeleton, and gene expression [27]. Moreover, GSK3β has been found to affect cell phenotypes like cell cycle, survival, proliferation, and apoptosis [28]. Previous studies have shown that GSK3β is upregulated or activated in CRC [29, 30]. Herein, we observed that the phosphorylation level of GSK-3β was suppressed in RKO cells when NUBP2 was knocked down, while upregulated upon NUBP2 overexpression. Notably, inhibiting GSK-3β led to cell cycle arrest and apoptosis in CRC [30]. Consistent with these findings, our findings demonstrated that treatment with CHIR-99021 HCl (10 μM), a GSK3 inhibitor, suppressed cell proliferation and facilitated apoptosis in CRC cells. What is more, the promoting effect of NUBP2 on the malignant behaviors of CRC cells could be reversed by the addition of CHIR-99021 HCl. Importantly, PI3K/AKT/GSK3β signaling pathway has been implicated in the EMT in CRC [17, 18]. Our study revealed that knocking down NUBP2 effectively inhibited EMT in CRC, while NUBP2 overexpression increased the expression of p-GSK-3β, p-AKT, p-PI3K, β-catenin and Cyclin D1. Interestingly, treatment with a GSK3 inhibitor partially reversed these effects, indicating that NUBP2 contributed to EMT and progression in CRC via GSK-3 regulation. However, further investigations are necessary to elucidate the precise mechanisms by which NUBP2 modulates EMT in CRC cells via the PI3K/AKT/GSK3β pathway.

## Conclusion

In conclusion, our findings revealed that NUBP2 knockdown repressed malignant phenotype of CRC cells by suppressing cellular proliferation and migration while stimulating apoptosis, and inhibited tumor growth in vivo. Furthermore, restoring GSK3β activity could reverse the malignant phenotype of CRC cells induced by NUBP2 overexpression. Overall, the current study emphasized the potential of NUBP2 as a novel therapeutic target for combating CRC progression, providing valuable insights into the underlying mechanisms of CRC progression.

## Supplementary Information

Below is the link to the electronic supplementary material.

Supplementary file1 (DOCX 799 KB)

## Data Availability

The data set supporting the results of this article are included within the article.
