# Supplementary material for: Abnormal upregulation of NUBP2 contributes to cancer progression in colorectal cancer
Source: Mol Cell Biochem. 2024 Mar 16;480(1):399–410. doi: 10.1007/s11010-024-04956-8 (PMC11695649; doi:10.1007/s11010-024-04956-8)
Supplement: Supplementary file 1 — Supplementary file1 (DOCX 799 KB) [file 11010_2024_4956_MOESM1_ESM.docx]

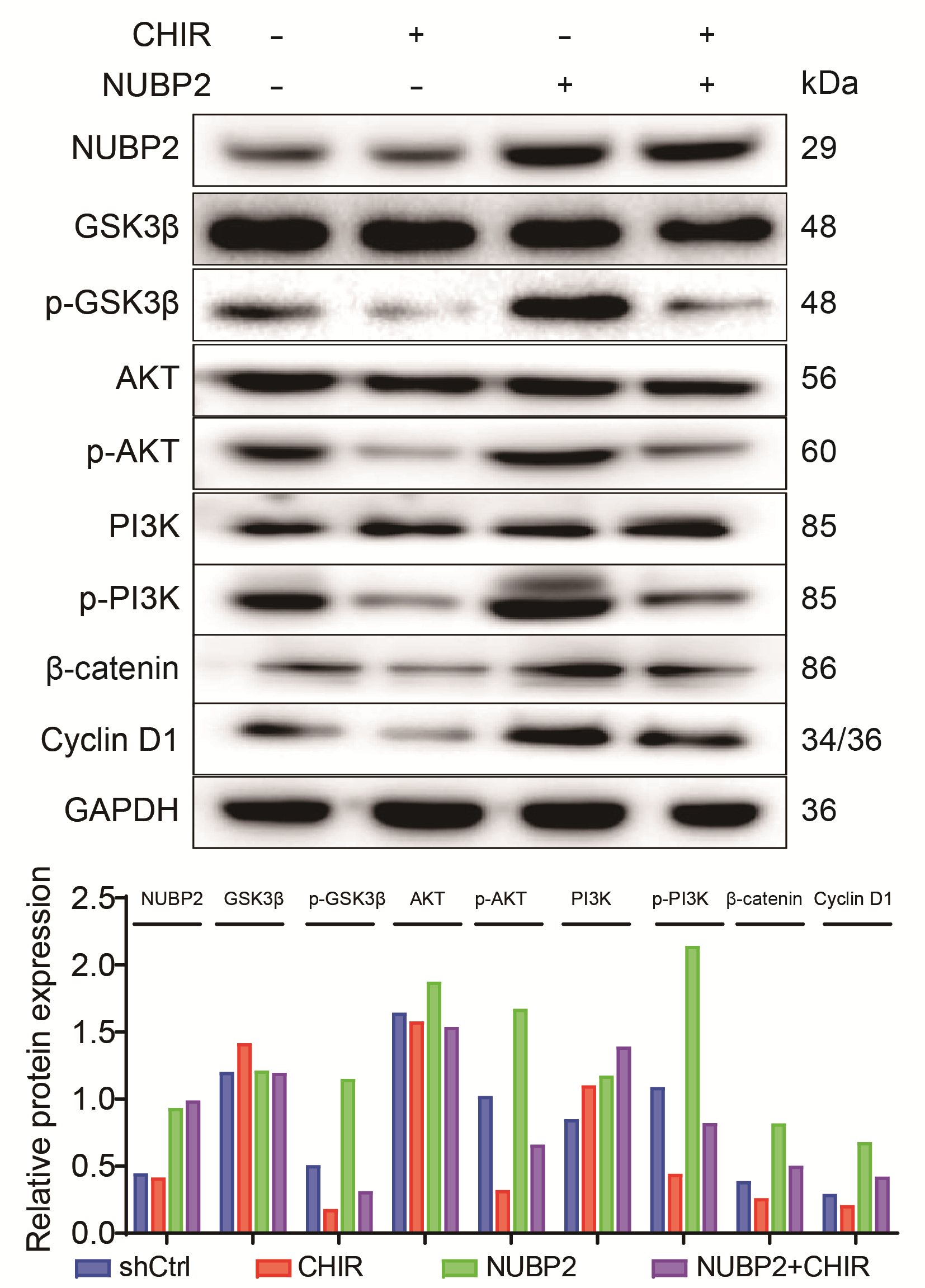


**Figure S1** The expression of proteins associated with the GSK3 pathway was analyzed by western blot in HCT116 cells with NUBP2 overexpression and CHIR-99021HCl treatment.

**Table S1** Antibodies applied in Western blot of this research.

| Primary antibody | Size/kDa | Diluted multiples | Source | Company | Catalog No. |
| --- | --- | --- | --- | --- | --- |
| NUBP2 | 29 | 1:500 | Rabbit | Proteintech | 15409-1-AP |
| E-cadherin | 97/125 | 1:3000 | Rabbit | Proteintech | 20874-1-AP |
| N-Cadherin | 100/130 | 1:1000 | Rabbit | Proteintech | 22018-1-AP |
| Vimentin | 51/57 | 1:2000 | Rabbit | Bioss | bs-8533R |
| Bax | 21 | 1:2000 | Rabbit | Proteintech | 50599-2-Ig |
| Bad | 18/23 | 1:2000 | Rabbit | Abcam | ab32445 |
| GSK3β | 48 | 1:2000 | Rabbit | Proteintech | 22104-1-AP |
| p-GSK3β | 48 | 1:1000 | Mouse | Proteintech | 67558-1-Ig |
| AKT | 56 | 1:3000 | Rabbit | Proteintech | 10176-2-AP |
| P-AKT | 60 | 1:1000 | Mouse | Proteintech | 66444-1-Ig |
| PI3K | 85 | 1:1000 | Rabbit | CST | 4257S |
| P-PI3K | 85 | 1:750 | Rabbit | Bioss | bs-3332R |
| GAPDH | 36 | 1:30000 | Mouse | Proteintech | 60004-1-lg |
| Secondary antibody |  | Diluted multiples |  | Company | Catalog No. |
| Goat Anti-Rabbit |  | 1:3000 |  | Beyotime | A0208 |
| Goat Anti-Mouse |  | 1:3000 |  | Beyotime | A0216 |
